# Comparative Biochemical Outcomes, Effectiveness and Tolerance of Densiron 68 and Oxane HD for the Management of Complicated Retinal Detachment

**DOI:** 10.4274/tjo.galenos.2019.24294

**Published:** 2019-12-31

**Authors:** Chafik Keilani, Edouard Augstburger, Mathieu Robin, Amélie Beaugrand, Raphaëlle Ores, José-Alain Sahel, Sarah Ayello-Scheer

**Affiliations:** 1 Quinze-Vingts National Hospital, Department of Ophthalmology IV / Sorbonne University, Faculty of Medicine Pierre-et-Marie-Curie/Assistance Publique-Hopitaux de Paris, Paris, France; 2 Quinze-Vingts National Hospital, Department of Ophthalmology III, Paris, France; 3 Quinze-Vingts National Hospital, Department of Biomedical Informatics, Paris, France; 4 Quinze-Vingts National Hospital, Department of Ophthalmology, Paris, France

**Keywords:** High-density silicone oils, Densiron® 68, Oxane® HD, complicated inferior retinal detachment, proliferative vitreoretinopathy

## Abstract

**Objectives:**

To compare biochemical outcomes, effectiveness, and tolerance of two high-density silicone oils (HDSOs), silicone oil- RMN3 (Oxane^®^ HD) and silicone oil-Densiron-68 (Densiron^®^ 68), for the management of complicated retinal detachment (RD) associated with inferior proliferative vitreoretinopathy (PVR).

**Materials and Methods:**

This was a retrospective, single-centre, comparative case series of 23 patients treated between September 2014 and June 2016. The main inclusion criteria were RD with inferior PVR receiving Oxane^®^ HD or Densiron^®^ 68 following pars plana vitrectomy. The main outcome measures were anatomical success, rate of RD recurrence, and best-corrected visual acuity (BCVA) at 6 months. Secondary outcomes were short-term complications.

**Results:**

Twenty-three eyes were included: 16 eyes with Densiron^®^ 68 tamponade and 7 eyes with Oxane^®^ HD tamponade. Anatomical success under HDSO was significantly higher in the Densiron^®^ 68 group (100%) than in the Oxane^®^ HD group (42.8%) (p=0.0455). Recurrent RD was observed in 42.8% of eyes under Oxane^®^ HD, but in none of the patients under Densiron^®^ 68 (p=0.001). Six months after surgery, mean BVCA values (+/- standard deviation) with Densiron^®^ 68 and Oxane^®^ HD were 0.83±0.62 logMAR and 1.81±0.65 logMAR, respectively. BVCA was significantly better in the Densiron^®^ 68 group (p=0.006). No significant differences were observed with regard to intraocular pressure, emulsification, or intraocular inflammation.

**Conclusion:**

Densiron^®^ 68 appears to be more effective than Oxane^®^ HD for the management of RD associated with PVR. A randomized, controlled, interventional study is needed to demonstrate this difference.

## Introduction

Silicone oil has been demonstrated to be an effective long-term internal tamponade agent for the treatment of superior breaks and retinal detachment (RD) complicated by proliferative vitreoretinopathy (PVR) and is currently widely used.^1,2,3,4^ Although lighter-than-water silicone oils can provide an effective tamponade effect for the superior retina, they provide little support for the inferior retina. This can result in the accumulation of fluid in the inferior quadrants of the retina during silicone oil tamponade and enhanced proliferation.^5^ The most frequent complication of the use of silicone oil as an internal tamponade is therefore the persistence or recurrence of inferior RD due to advanced PVR.^6,7,8,9,10^ In the 1990s, high-density (> 1.90 g/cm^3^) perfluorocarbons and partially fluorinated alkanes were used for RD surgery. However, these fluids were associated with tamponade emulsification, intraocular inflammation, and rises in intraocular pressure (IOP).

At the beginning of the 21^st^ century, the first high-density silicone oils (HDSO) were introduced as endotamponade agents for RD with inferior PVR. HDSOs are a mixture of silicone oil and partially fluorinated alkanes and have a relative density of 1.02–1.06 g/cm^3^ at 22°C. The mixtures were developed to decrease postoperative tamponade emulsification, intraocular inflammation, and rises in IOP.

**Figure 1:**
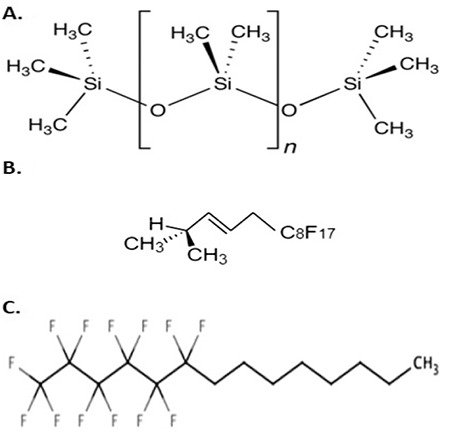
Chemical structure of HDSO components, A. Polydimethylsiloxane, B. RMN3, C. Perfluorohexyloctane

In 2003, a silicone oil–RMN3 mixture, Oxane^®^ HD, which has a density of 1.03 g/cm^3^ at 22°C and a viscosity of 3300 centistokes (cSt) at 25°C was introduced. Oxane HD^®^ consists of 11% RMN3 and 89% 5700 cSt silicone (Oxane 5700^®^), a polydimethylsiloxane (Figure 1A), and RMN3 (Figure 1B). It is a hydrogenated and fluorinated olefin, 1 perfluorooctyl-5-methylhex-2-ene and is soluble in silicone oil. It was followed in 2005 by another HDSO, a silicone oil–perfluorohexyloctane mixture called Densiron^®^ 68, with a density of 1.06 g/cm^3^ at 22*°C* and a viscosity of 1400 cSt at 25°C. Densiron^®^ 68 comes from a blend of 69.5% 5000 cSt silicone (Siluron 5000^®^), a polydimethylsiloxane, and 30.5% of perfluorohexyloctane (F6H8^®^) (Figure 1C).

These HDSOs have shown higher attachment rates in complicated RD.^11,12,13,14,15,16,17^ A recent meta-analysis has shown that the complication spectrum of these new-generation HDSOs seems to be similar to that of conventional silicone oil tamponades.^18^ These two approved heavier-than-water silicone oil tamponades, Oxane^®^ HD and Densiron^®^ 68, are commonly used in clinical practice.

Densiron^®^ 68 has a higher density than Oxane^®^ HD. Nevertheless, due to its lower viscosity, Densiron^®^ 68 could result in more tamponade emulsification, intraocular inflammation, and IOP elevation than Oxane^®^ HD. In the present study, we compared the effectiveness and tolerance of Densiron^®^ 68 and Oxane^®^ HD for the management of complicated inferior RD in order to provide a decision-making table for vitreoretinal surgeons for the use of HDSO.

## Methods

We retrospectively reviewed the medical records of patients with complicated RD treated using Oxane^®^ HD or Densiron^®^ 68 as an internal tamponade after pars plana vitrectomy (PPV) between September 2014 and June 2016. Prior to surgery, all patients underwent complete ophthalmologic evaluation including detailed slit-lamp and funduscopic examination, assessment of best corrected visual acuity (BCVA), and tonometry. The diagnosis of complicated RD was based on funduscopic examination of the retina. B-scan ultrasonography was performed if RD was difficult to assess using funduscopic examination. Inclusion criteria were: complicated RD, age over 18 years, pseudophakic eye, surgery performed under peribulbar anesthesia, patient able to communicate effectively, and informed consent obtained from the patient. Complicated RD was defined as follows: RD secondary to PVR and to inferior or posterior tears. In addition, recurrent and chronic RD was considered complicated RD. PVR was assessed according to the updated Retina Society Classification. Exclusion criteria were: chronic glaucoma, diabetes with retinopathy or diabetic maculopathy, active periocular or ocular infection, diagnosis of uncontrolled systemic disease, epiretinal membrane, non-French speakers, hallucinations, delirium, and Alzheimer’s disorders. All surgeries were carried out by three specialist retinal surgeons at the Centre Hospitalier National d’Ophtalmologie (CHNO) des Quinze-Vingts, Paris, France.

Two HDSO were used: Oxane^®^ HD (Bausch & Lomb, Germany) and Densiron^®^ 68 (Fluoron Gmbh, Germany). The use of Oxane^®^ HD or Densiron^®^ 68 was based on surgeon preference. Both Oxane^®^ HD and Densiron^®^ 68 were used by the three surgeons.

All surgeries consisted of standard three-port 23-gauge pars plana vitrectomy. Membrane peeling, retinotomy, and retinectomies were performed if needed. Retinal breaks were treated by endolaser. In patients with previous injected standard silicone oil, this agent was removed first. PFCL (perfluorocarbon liquid) was used during surgery only if retinotomy was performed. Subconjunctival injection of 4 mg dexamethasone was performed at the end of surgery. Antibiotic–steroid eye drops were applied 4 times daily for 1 month after surgery. HDSO was removed by active aspiration using an 18-gauge cannula and two 23-gauge ports for endoillumination probe and infusion.

Patients were examined at 1 week and at 1, 3, and 6 months postoperatively. Functional outcomes such as anatomical success under HDSO and after HDSO removal, BCVA, IOP changes, emulsification, intraocular inflammation scored with ocular inflammation grading scale (Table 1) and complications during and after endotamponade removal were documented at each follow-up visit. Functional outcomes were assessed via funduscopic examination and intraocular inflammation was assessed using slit-lamp examination.

Collected data included patient demographics, indication for pars plana vitrectomy, procedure performed, type of HDSO, and duration of follow-up.

The primary endpoints were anatomical success defined as a completely attached retina under HDSO, time under HDSO, rate of RD recurrence under HDSO tamponade in the Oxane^®^ HD and Densiron^®^ 68 groups, and mean BCVA at postoperative 6 months. In addition, data were compared between the Oxane^®^ HD and Densiron^®^ 68 groups during the same period in our hospital. Secondary endpoints were rate of RD recurrence after HDSO removal, mean duration to RD recurrence after HDSO removal, IOP changes, emulsification, intraocular inflammation, and endophthalmitis.

### Statistical Analysis

BVCA values were converted into logMAR units. Primary and secondary endpoints were evaluated using chi-square and Mann–Whitney U tests, with p < 0.05 considered statistically significant. The nonparametric Mann–Whitney test is widely used to test treatment effects by comparing the outcome distributions between two groups. Source apportionments of elements were carried out using MS Excel 2010^®^. Data were analyzed using XLSTAT^®^ software. The Quinze-Vingts ethics committee approved this study.

## Results

**Figure 2:**
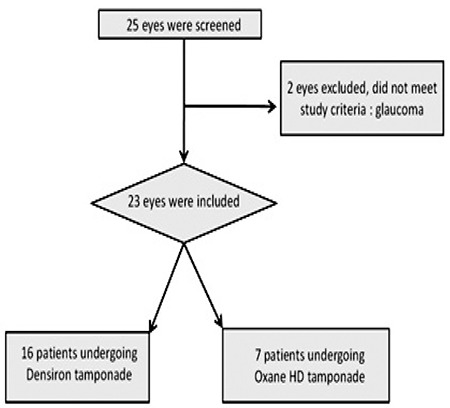
Study flowchart

Twenty-three eyes of 21 patients were included: 16 eyes treated with Densiron^®^ 68 tamponade and 7 eyes with Oxane^®^ HD tamponade. Two patients with glaucoma were excluded (Figure 2). Analysis of the patient demographics (Table 2) showed that the average age was 65 ± 12 years (mean ± standard deviation). No significant difference in age was observed between the Densiron^®^ 68 and Oxane^®^ HD groups. In addition, no differences were found for any of the patient demographics, such as age or ethnicity, nor for anesthesia (Table 2). The indications for HDSO tamponade were similar for both groups and included: RD secondary to PVR with inferior breaks. The mean follow-up time was similar for both groups: 9 ± 4 months.

The prevalence of macular detachment before surgery was approximately 80% in both the Densiron^®^ 68 and the Oxane^®^ HD group (Table 2). Two patients in the Densiron^®^ 68 group were under standard silicone oil tamponade before inclusion. Four patients had undergone vitrectomy with gas endotamponade (three in the Densiron^®^ 68 group and two in the Oxane^®^ HD group). No eyes in eithergroup had phacoemulsification before vitrectomy. All eyes already had an intraocular lens (IOL) before surgery. Retinectomies and retinotomies were only performed on patients with PVR. The rate of retinotomy was similar in both groups (Table 2). No statistically significantly difference in BVCA was observed between the Densiron^®^ 68 group and the Oxane^®^ HD group prior to surgery (p = 0.20) (Table 2).

### Anatomical Success and RD Recurrence Under HDSO

Anatomical success under HDSO was significantly higher in the Densiron^®^ 68 group (100%) than in the Oxane^®^ HD group (42.8%) (p = 0.0455). Of the 7 eyes treated using Oxane^®^ HD, 3 (42.8%) showed recurrent RD with macular detachment under HDSO. All of these detachment events occurred in the inferior retinal quadrants. None of the 16 eyes in the Densiron^®^ 68 group showed recurrent RD under HDSO. Anatomical success after HDSO removal was also significantly higher in the Densiron^®^ 68 group (81.3%) than in the Oxane^®^ HD group (48%) (p = 0.001). In the Densiron^®^ 68 group, the mean duration until silicon oil removal was 4.18 ± 2.56 months compared with 3.50 ± 2.20 months in the Oxane^®^ HD group. The mean duration until silicon oil removal was shorter in the Oxane^®^ HD group due to recurrent RD which occurred after 1 month in 2 eyes and only 1 week in 1 eye in this group (Table 3).

### Rate of RD Recurrence After HDSO Removal

Concerning the secondary endpoints, of the 16 eyes in the Densiron^®^ 68 group, 3 (18.7%) displayed recurrent RD with macular detachment following HDSO removal. All of these detachment events occurred in the inferior retinal quadrants. The mean duration until recurrent RD was 5 months after Densiron^®^ 68 removal. In the Oxane^®^ HD group, 3 of the 7 eyes displayed RD under HDSO. Recurrent RD was higher in the Oxane^®^ HD group than in the Densiron^®^ 68 group after HDSO removal (Table 3).

### BVCA at Postoperative 6 Months

Concerning the primary endpoints, mean BVCA at postoperative 6 months was 0.83 ± 0.62 logMAR in the Densiron^®^ 68 group and 1.81 ± 0.65 logMAR units in the Oxane^®^ HD group (Table 4). BVCA at 6 months was significantly better in the Densiron^®^ 68 group than in the Oxane^®^ HD group (p = 0.006) (Table 4). The Densiron^®^ 68 group showed a significant improvement in BVCA at postoperative 6 months compared to before surgery (0.83 ± 0.62 logMAR vs. 1.48 ± 0.78 logMAR; p = 0.01), while the postoperative change in BCVA was not statistically significant in the Oxane^®^ HD group (1.97 ± 0.48 logMAR vs. 1.81 ± 0.65 logMAR; p = 0.80) (Table 4).

### Tolerance Under HDSO

**Figure 3:**
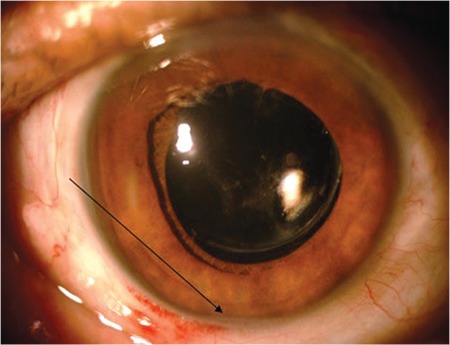
Inferior emulsification under Densiron^®^ 68 tamponade in anterior chamber (black arrow)

**Figure 4:**
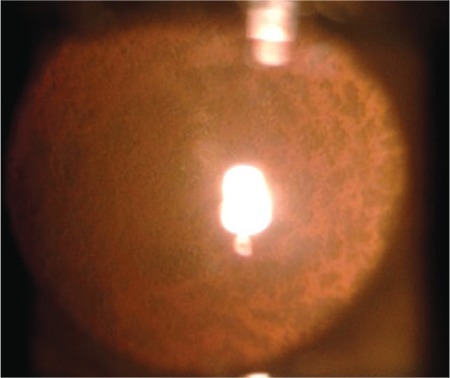
Retrocapsular emulsification under Densiron^®^ 68 tamponade

The Densiron^®^ 68 and Oxane^®^ HD groups were compared in terms of IOP changes, emulsification, and intraocular inflammation (Table 5). No significant differences were observed between the two groups. Densiron^®^ 68 was well tolerated with the exception of one patient who developed chronic glaucoma. Both HDSOs induced intraocular inflammation, but only in a few patients, about 10% in both groups. All patients that had inflammation were assessed as grade 2 on the ocular inflammation grading scale. Several cases of HDSO emulsification were observed: 25% in Densiron^®^ 68 group and 16% in Oxane^®^ HD group after 1 month (Figures 3 and 4). No significant differences were observed between the two groups for emulsification. No cases of endophthalmitis were noted in either group.

### Surgeon Use of HDSOs

Use of Densiron^®^ 68 and Oxane^®^ HD was similar for all of the surgeons. In addition, RD recurrence after HDSO removal was similar for each surgeon (Table 6).

## Discussion

The use of HDSO is required for internal tamponades when the duration of the tamponade is greater than one month and when there is a significant risk of recurrent RD without internal tamponade. The use of HDSO is therefore strongly indicated for patients with complex retinal detachments, as all patients under Densiron^®^ 68 had complete anatomical success.

In this study, we compared the effectiveness and tolerance of Densiron^®^ 68 and Oxane^®^ HD for the management of complicated RD. To the best of our knowledge, no studies have yet compared these two HDSOs.

We found a statistically significant difference between the Densiron^®^ 68 and Oxane^®^ HD groups in terms of anatomical and functional success rates. Our final 81.3% success rate for primary reattachment at least 6 months after Densiron^®^ 68 removal is consistent with the results of other studies.^19,20^ Only 48% of patients had anatomical success after Oxane^®^ HD removal. This suggests that Densiron^®^ 68 provided better support for the inferior retina than Oxane^®^ HD. Densiron^®^ 68 is a mixture of 30.5% perfluorohexyloctane (F6H8) and 69.5% polydimethylsiloxane (SiO) (vol/vol). The specific gravity of Densiron^®^ 68 (1.06 g/cm^3^ at 25°C) is higher than that of Oxane^®^ HD (1.03 g/cm^3^ at 25°C). Because a heavy intraocular tamponade is needed for the management of complicated RD, the greater density of Densiron^®^ 68 could explain the lower rate of retinal redetachment under Densiron^®^ 68 compared with Oxane^®^ HD.

In addition, our result of 81.3% anatomical success under Densiron^®^ 68 HD is similar to other studies.^21^

All redetachment events were observed in the inferior retina. Densiron^®^ 68 and Oxane^®^ HD could also be effective in providing support for the superior retina, as no superior RD events or tears were observed following HDSO removal.

In addition, patients who received an endotamponade with Densiron^®^ 68 showed more favorable functional outcomes, with mean postoperative 6-month BCVA that was significantly better compared to preoperative BCVA (p = 0.01) and the postoperative 6-month BCVA in patients treated with Oxane^®^ HD (p = 0.006). The rate of functional success was disappointing in patients who received an endotamponade with Oxane^®^ HD because of anatomical failure due to the high RD recurrence rate (42.8%).

In the present study, the mean time to Densiron^®^ 68 removal was 4.18 ± 2.56 months, which is relatively short. This could probably be explained by the fact that surgeons wanted to avoid potential complications associated with Densiron^®^ 68, especially emulsification. In the Densiron^®^ 68 group of this retrospective study, recurrent RD was observed in 18.7% eyes following HDSO removal. However, given the low complication rate in patients undergoing tamponade with Densiron^®^ 68, it is possible that a longer tamponade duration, for example 6 months, could decrease the RD recurrence rate following HDSO removal despite the general consensus that the timing of silicone oil removal has no impact on redetachment rate.^22^

Both groups showed similar complications, such as a rise in IOP, inflammatory reactions, and emulsification of HDSO, and complication rates did not differ significantly between the two groups. Similar complications have been observed in previous reports on the use of Densiron^®^ 68.^23,24,25^ One patient in the Densiron^®^ 68 group developed chronic glaucoma. Apart from this single case, Densiron^®^ 68 was well tolerated. Previous studies have reported that the use of Densiron^®^ 68 results in higher IOP levels, and more cases of inflammatory reaction and emulsification than Oxane^®^ HD because of its lower viscosity (1400 cSt for Densiron^®^ 68 vs. 3300 cSt for Oxane^®^ HD at 25°C.^26,27,28^ Nevertheless, in our study Densiron^®^ 68 and Oxane^®^ HD had similar, low complication rates. Two steps of the procedure could explain these low complication rates: first, all patients received a subconjunctival injection of 4 mg dexamethasone at the end of surgery, which reduces postoperative inflammation, and second, the ocular cavity was completely filled with HDSO. The importance of completely filling the ocular cavity with silicone oil has previously been demonstrated because the presence of an incomplete bubble promotes emulsification.^29^ In addition, the lower viscosity of Densiron^®^ 68 makes both injection and removal easier. Recently, Densiron^®^ Xtra has been produced. It has a lower viscosity (1200 cSt) than Densiron^®^ 68, allowing easy injection, especially with 25-gauge systems. It also allows easy removal and has a low emulsification rate.^30^

Many studies have studied retinal and corneal toxicity. No histological changes have been reported in rabbit or pig. Some lesions have been observed on optical microscopy, including minor mononuclear inflammatory reaction, some disorganization of the intercellular space between photoreceptors, nuclear densification in the outer nuclear layer, irregularities in the external limiting membrane, and intracellular edema in the outer retinal layers. In electron microscopy, intercellular edema at the level of the outer layer and disorganization of the inter-photoreceptor space have been well described, while the structure of the photoreceptor segments remained normal.^31,32,33,34,35^

Limitations of the present study are the lack of a randomized procedure and the small number of patients in the Oxane^®^ HD group. Surgical steps during the procedure were similar for the two groups, which reduces bias in evaluating success after surgery. This study is retrospective with low-level evidence; however, our results for anatomical success and mean BVCA in patients after HDSO tamponade could guide future statistical considerations if a randomized controlled study is to be conducted. The use of Oxane^®^ HD or Densiron^®^ 68 was based on surgeon preference, which is another source of bias.

One of the main advantages of using HDSOs is that patients are not required to lie face-down after surgery, a position that can be difficult to maintain, especially with older patients.

In this study, all retinal surgeries were performed by three surgeons, which could have introduced bias. However, the procedures were similar and both Oxane^®^ HD and Densiron^®^ 68 were used by the three surgeons, so any bias should be minimal. Moreover, RD recurrence after HDSO removal was similar for each surgeon (Table 6).

Our study suggests that Densiron^®^ 68 is a better endotamponade agent than Oxane^®^ HD for treating complicated inferior RD due to its higher density. Thus, we propose a decision-making table for the management of complicated inferior RD (Table 7).

## Conclusion

In summary, Densiron^®^ 68 seems to be more effective for endotamponade than Oxane^®^ HD to manage complex RD associated with PVR. Densiron^®^ 68 provides high anatomical and functional success rates. The rate of complications with Densiron^®^ 68 is low with adapted postoperative management. A controlled, randomized interventional study is now required to further investigate the advantages of Densiron^®^ 68 and demonstrate its superiority.

## Figures and Tables

**Table 1:**
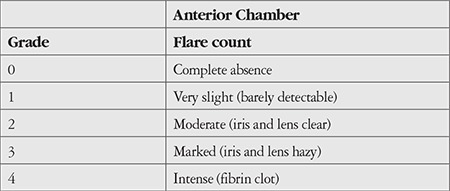
Ocular inflammation grading scale

**Table 2:**
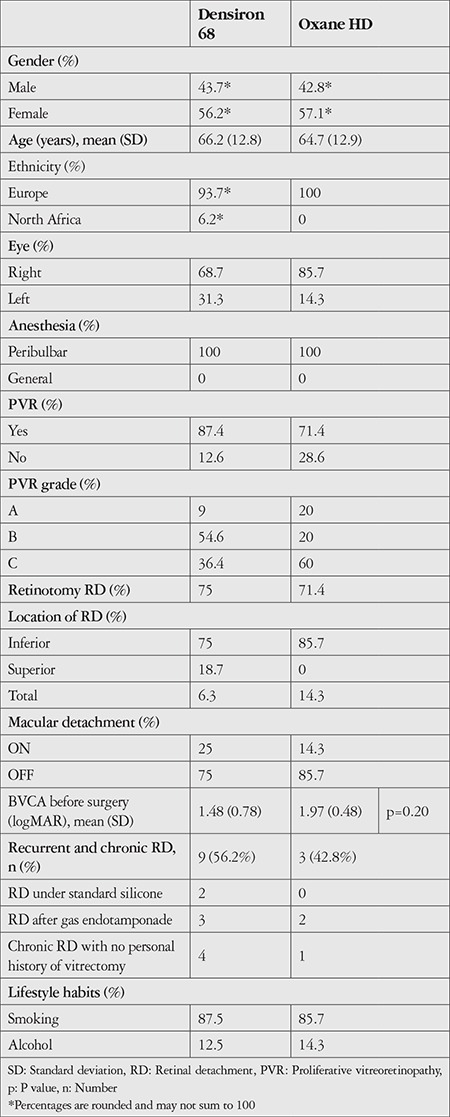
Study population characteristics

**Table 3:**
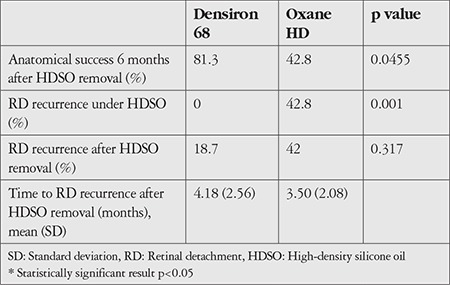
Anatomical success and retinal detachment recurrence under HDSO

**Table 4:**
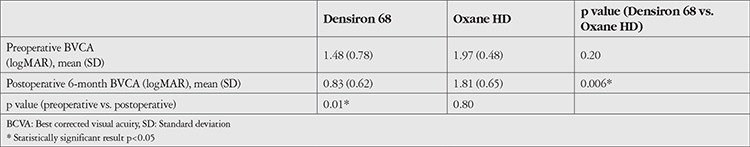
Best corrected visual acuity before and 6 months after surgery

**Table 5:**
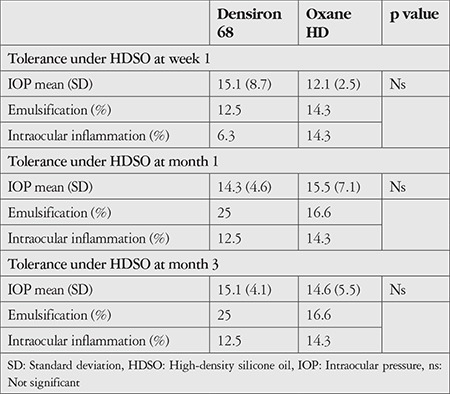
Tolerance under HDSO

**Table 6:**
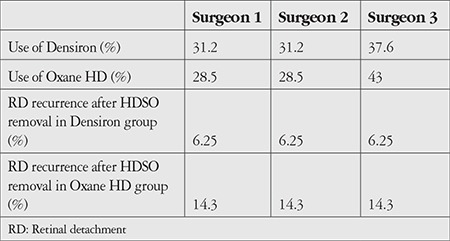
Use of HDSO for each surgeon

**Table 7:**
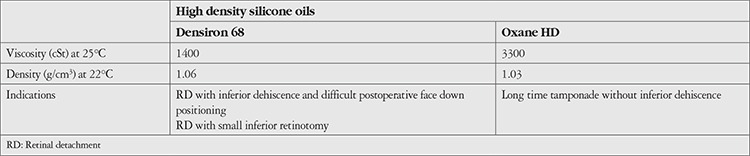
Proposition for management of inferior RD with HDSO
